# Crystal structure of the sodium salt of mesotrione: a triketone herbicide

**DOI:** 10.1107/S2056989024001439

**Published:** 2024-02-16

**Authors:** Olha Bereziuk, Kateryna Gubina, Viktor Trush, Vladimir Ovchynnikov

**Affiliations:** a National Taras Shevchenko University, Department of Chemistry, 01601 Kyiv, Volodymyrska str. 64, Ukraine; Harvard University, USA

**Keywords:** mesotrione, herbicides, sodium salt, crystal structure, TGA analysis

## Abstract

The crystal structure of the sodium salt of mesotrione, namely, *catena*-poly[[sodium-μ_3_-2-[(4-methane­sulfonyl-2-nitro­phen­yl)carbon­yl]-3-oxo­cyclo­hex-1-en-1-olato] ethanol monosolvate], {[Na(C_14_H_12_NO_7_S)]C_2_H_5_OH}_*n*_, is described. The X-ray structural analysis results reveal that the coordination sphere is established by two chelating O atoms, the O atom of the coordinated ethanol mol­ecule, and an O atom from the methyl­sulfonyl group of a neighboring mol­ecule. Simultaneously, an O atom of the cyclo­hexane fragment serves as a bridge to a neighboring sodium ion, forming a flat Na–O–Na–O quadrangle, thereby forming a mono-periodic polymer. The structure displays O—H⋯O hydrogen bonds and C—H⋯O short contacts. Thermogravimetric analysis (TGA) data indicate that the sodium salt of mesotrione decomposes in four stages.

## Abstract 2

The crystal structure of the sodium salt of mesotrione [2-(4-methyl­sulfonyl-2-nitro­benzo­yl)cyclo­hexane-1,3-dione] is described. A one-dimensional polymer is formed by the coordination of all functional groups except the NO_2_ group. The coordination number of the sodium atom in the compound is 5.

## 1. Chemical context

Mesotrione, 2-(4-methyl­sulfonyl-2-nitro­benzo­yl) cyclo­hexane-1,3-dione, is an organic compound classified as a triketone herbicide that is widely used in modern agriculture to control weeds and increase crop yields of corn (Mitchell *et al.*, 2001 ▸). The coordination properties of triketone herbicides are dictated by the presence of three ketone functional groups, which act as ligands, forming stable coordination complexes with metal ions such as Cu^2+^, Co^2+^ and Fe^3+^ (Le Person *et al.*, 2016 ▸). The stability of the chelates depends largely on the pH, as mesotrione is a weak acid that dissociates from the mol­ecular to the anionic form at higher pH, which is more resistant to hydrolysis and photolysis processes (Reynolds *et al.*, 2007 ▸). For a comparative study, the crystal structure of the sodium salt of mesotrione, Na*L*, as well as analogues structures were retrieved from the Cambridge Structural Database (CSD, vesion 5.44, update of September 2023; Groom *et al.*, 2016 ▸) and their geometries and confirmations are discussed (Kang *et al.*, 2015 ▸); Hou *et al.*, 2010 ▸; Wu *et al.*, 2002 ▸). 
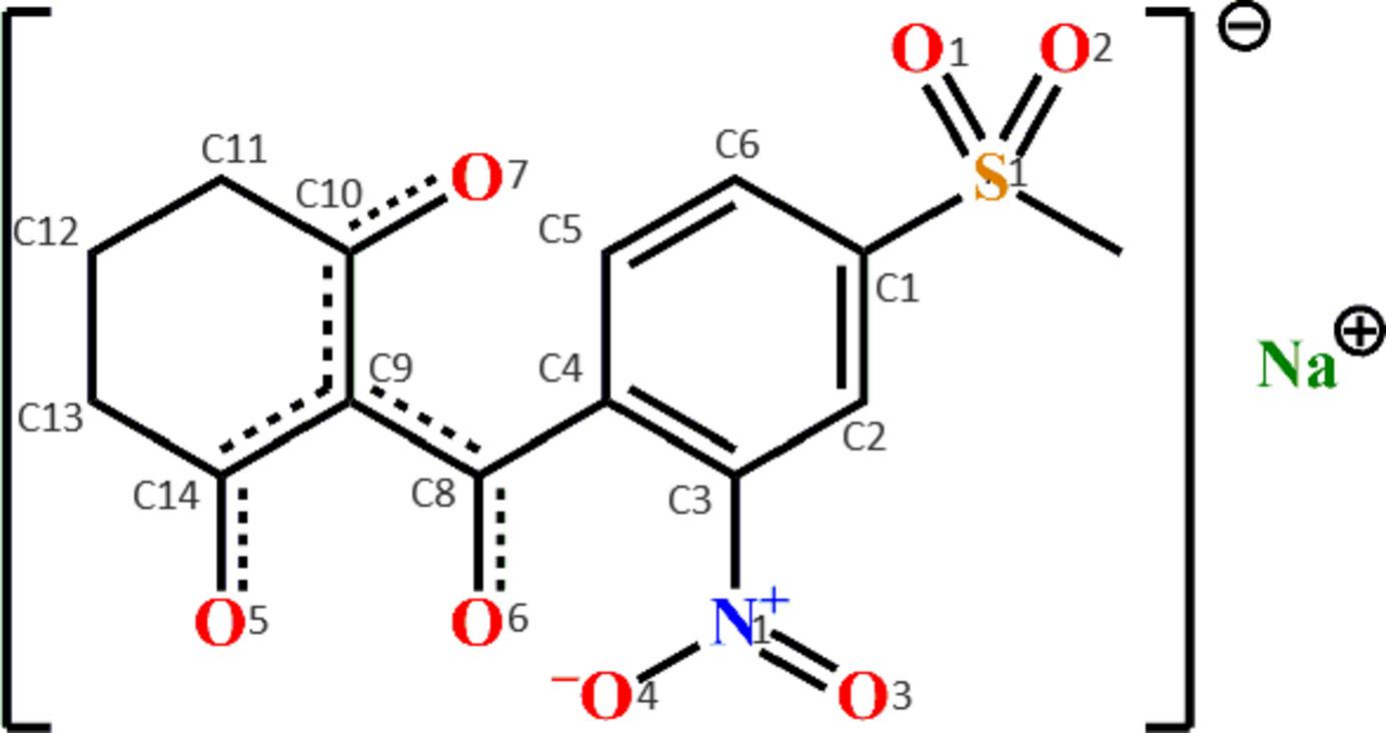


## 2. Structural commentary

**Figure 1:**
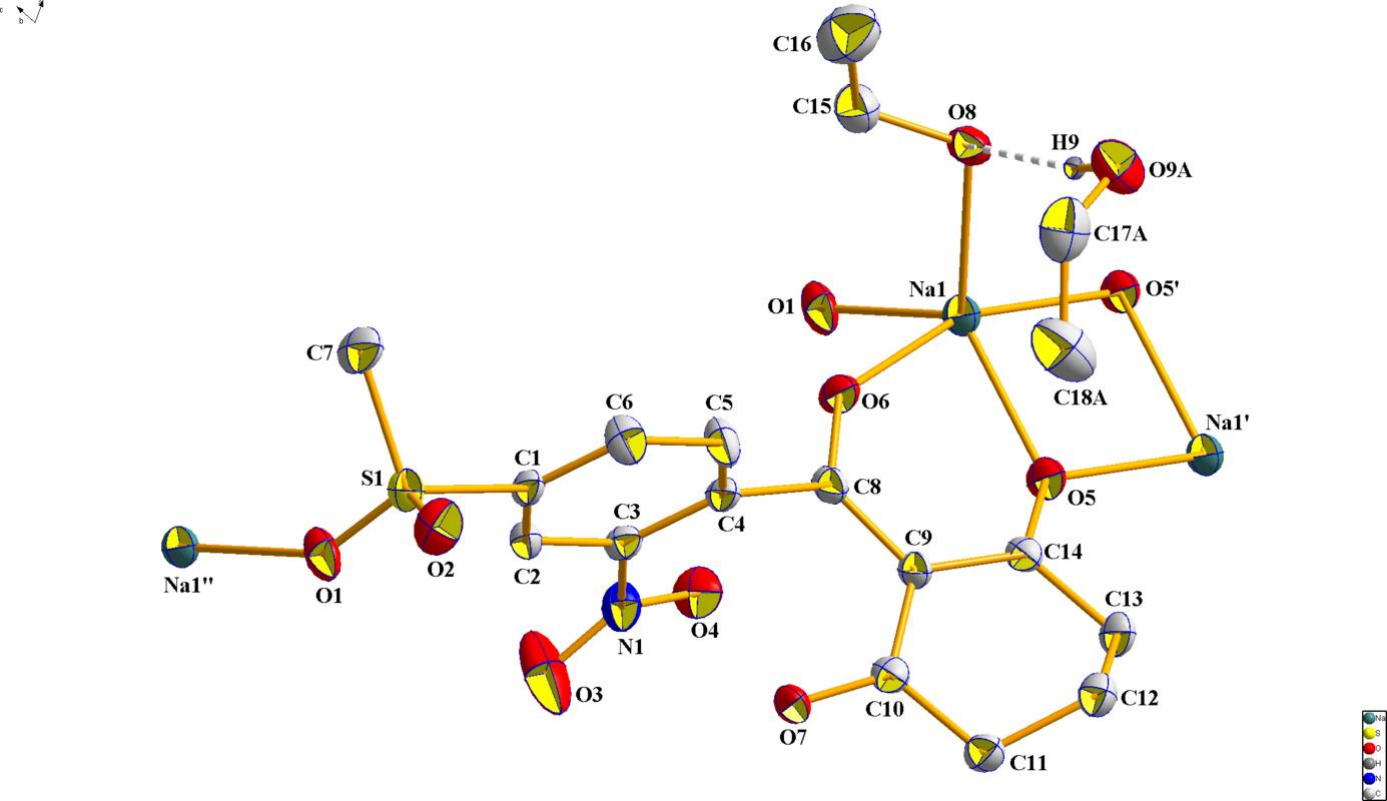
The fragment of the structure of the sodium salt of mesotrione, showing the atom-numbering scheme for non-hydrogen atoms and displacement ellipsoids at 50% probability level.

Selected geometrical parameters of the sodium salt of mesotrione are summarized in Table 1 ▸. The ligand shows a polydentate function. Coordination to the sodium ion occurs through the formation of a 6-membered chelate involving two oxygen atoms from the two keto groups (Fig. 1 ▸). This leads to the occurrence of π-conjugation within the chelate ring, leading to a shortening of the C—C bonds by 0.06 (3) Å and lengthening of C=O bonds by 0.062 (3) Å in comparison to the free ligand H*L* (Table 2 ▸). In turn, in the mesotrione sodium salt, the occurrence of conjugation in the triketonate ligand results in a decrease in the conjugation between the benzene ring and the chelate ring, as evidenced by a 0.014 (3) Å increase in the C4—C8 bond length (Table 2 ▸).

**Figure 2:**
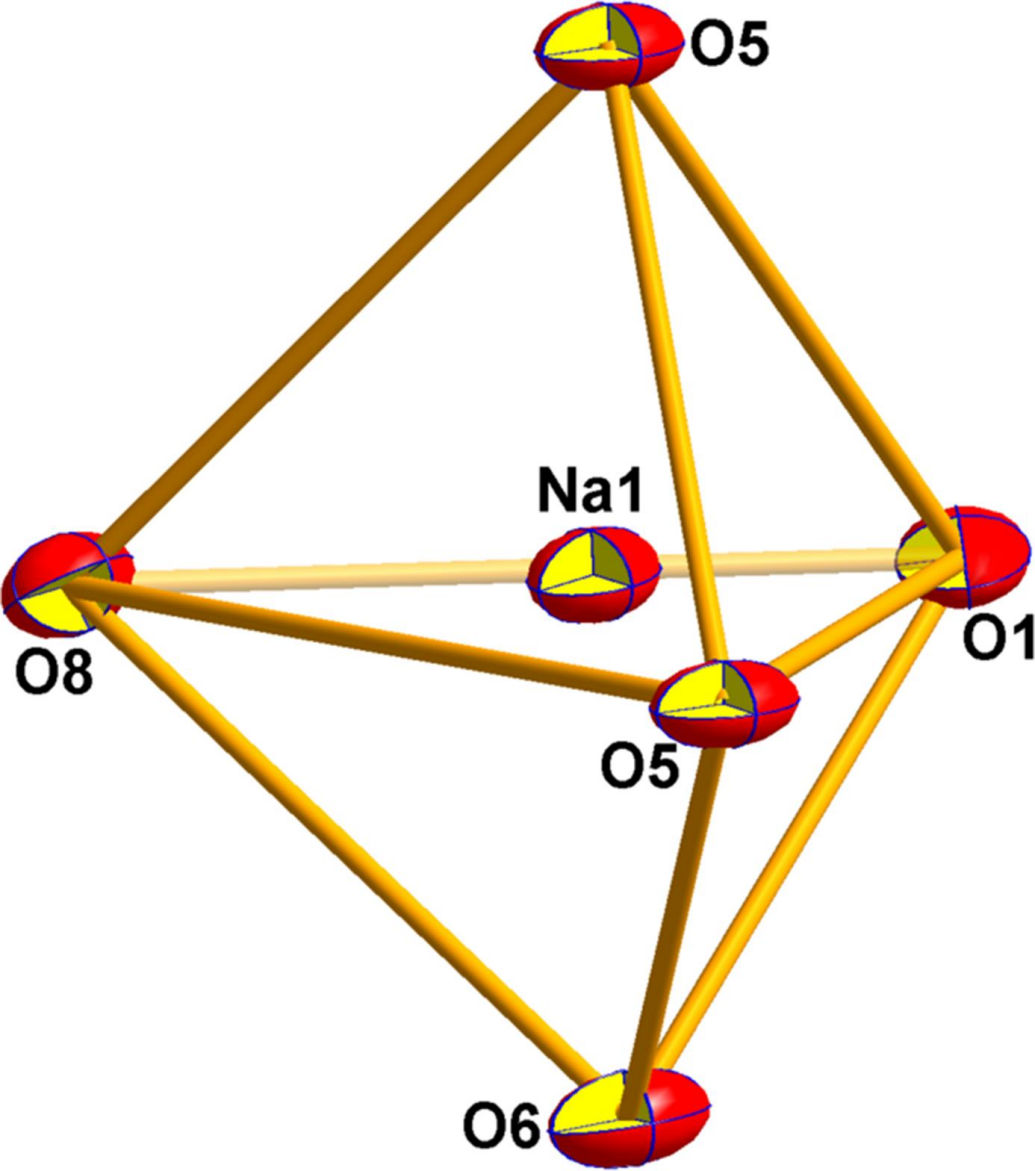
Coordination polyhedron of the sodium salt of mesotrione.

The chelate fragment tends towards a planar structure. Simultaneously, the oxygen atom O5 of the cyclo­hexane fragment serves as a bridge to a neighboring sodium ion, forming a flat quadrangle Na1–O5–Na1^i^–O5^i^ constituting the linker that forms the polymer chain (Fig. 2 ▸).

The benzene and cyclo­hexane ring conformations in the structure of sodium salt and free ligand are similar. The benzene ring has a planar conformation, while the cyclo­hexane ring represents a *semi chair* with a bend in the line linking atoms C11–C13. The main geometrical characteristics of hydrogen bonds of the compound [Na*L*(EtOH)]·EtOH are given in Table 3 ▸.

The environment sphere of the sodium ion comprises the oxygen atoms O5 and O6 of the chelate, the bridging oxygen atom O5^i^, the oxygen atom O8 from the coordinated ethanol mol­ecule, and the oxygen atom O1^ii^ from the methyl sulfonyl group of a neighboring mol­ecule (Fig. 2 ▸). Using the *SHAPE* program (Version 2.1; Llunell *et al.*, 2013 ▸), it was determined that the environment of the sodium atom is close to *D*_3*h*_ symmetry (trigonal bipyramid) with a convergence factor of 1.6.

## 3. Supra­molecular features

**Figure 3:**
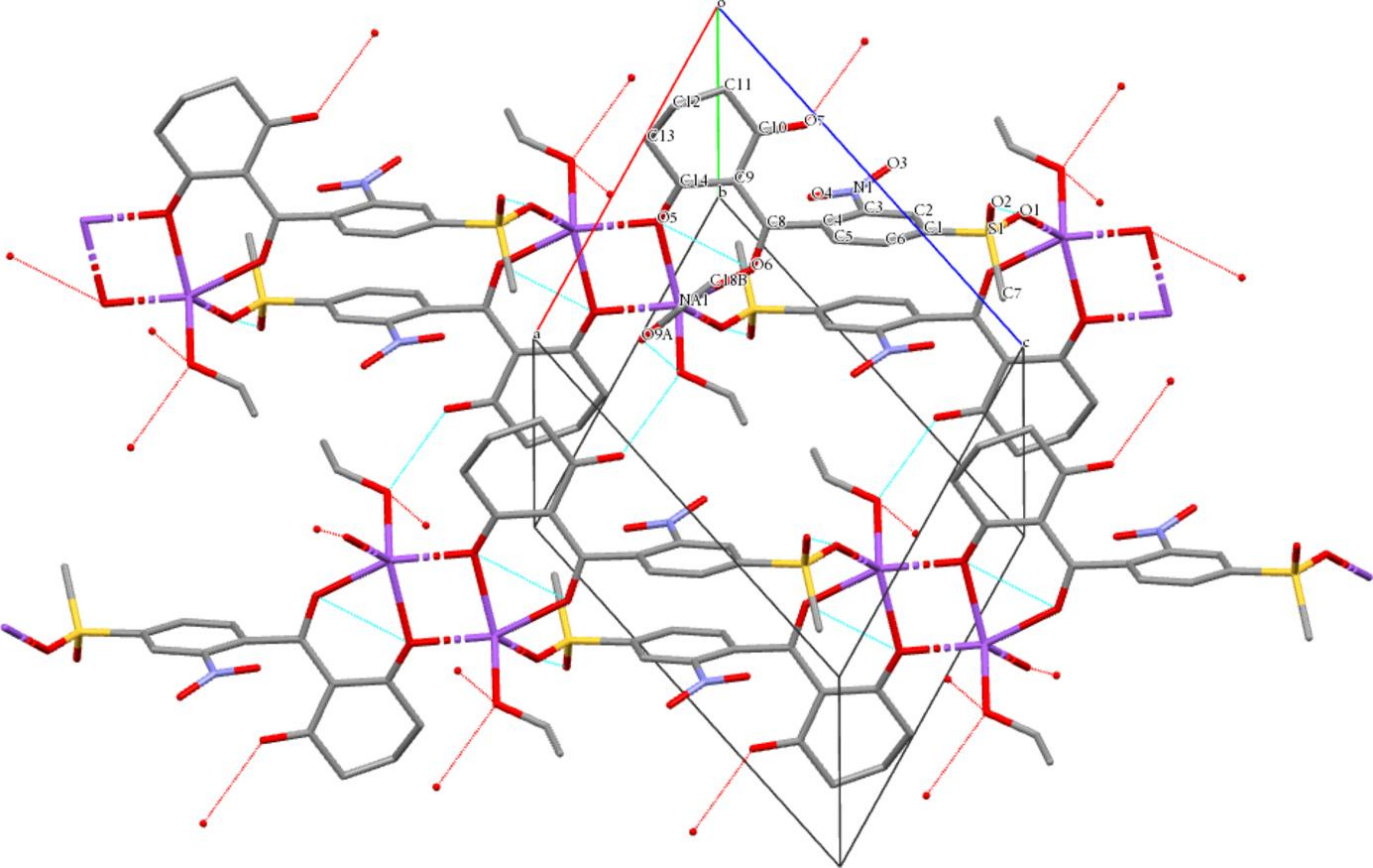
Crystal packing in a cell with projection onto the *ac* plane. Hydrogen bonds are highlighted in blue.

In the crystal structure of the sodium salt of mesotrione, the mol­ecules are assembled in a polymer chain (Fig. 3 ▸). Two types of hydrogen bonds are observed: the first between the oxygen atom of the uncoordinated ethanol mol­ecule (O9*A*) and the oxygen atom (O8) of the coordinated ethanol mol­ecule [2.870 (4) Å] and the second between the oxygen atom (O8) of a coordinated ethanol mol­ecule and the free oxygen atom (O7) of the keto group of a neighboring mol­ecule not involved in coordination [2.681 (2) Å]. In the structure of the coordination compound, three types of short contacts are observed, *viz*. C2—H2⋯O6^ii^ [3.229 (3) Å], C7—H7*B*⋯O4^ii^ [3.200 (3) Å], and C7—H7*C*⋯O9*A* ^iv^ [3.356 (4) Å] (symmetry codes are as per Table 3 ▸).

## 4. Experimental

The FT–IR spectra of the solids were recorded in a KBr matrix in the range 4000–400cm^−1^ using a Perkin-Elmer Spectrum BX2 spectrometer. ^1^H NMR spectra were recorded using a WR-400 Bruker NMR spectrometer at room temperature in DMSO-*d^6^*, with TMS used as the inter­nal standard. Studies on the thermal properties of the sodium salt of mesotrione were conducted using a synchronous TG/DTA analyzer, the Shimadzu DTG-60H. The sample was heated in an air atmosphere to 600°C in aluminum crucibles at a heating rate of 10°C min^−1^.

## 5. Synthesis and crystallization

Mesotrione was obtained commercially. Other chemicals and solvents used in this study were purchased from Aldrich and used without further purification.

**Figure 4:**
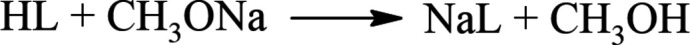
Synthesis of the sodium salt of mesotrione.

The sodium salt was prepared as shown in Fig. 4 ▸, where 2-(4-methyl­sulfonyl-2-nitro­benzo­yl)cyclo­hexane-1,3-dione was added to a freshly prepared sodium methyl­ate solution. For the monovalent metal sodium, the molar ratio of mesotrione to metal ions is 2:1. The resulting mixture was filtered, and the solvent was removed under vacuum. The yellowish crystalline powder (80% yield) was dissolved in a mixture of ethanol and methanol under heating (∼333 K) and then cooled to room temperature. After a while (∼72 h), monocrystals of the sodium salt of mesotrione, which were suitable for X-ray analysis, were formed.

[Na*L*(EtOH)]·EtOH: **IR** (KBr, cm^−1^): 1642 [ν_as_(C=O)_keto_], 1582 [ν_s_(C=O)_enol_], 1524 [ν_as_(NO_2_)], 1328 [ν_s_(NO_2_)], 1312 [ν_as_(SO_2_)], 1148 [ν_s_(SO_2_)].

[Na*L*(EtOH)]·EtOH: **NMR ^1^H** (400 MHz, DMSO-*d* ^6^, 298 K, TMS): Δ = 1.75 ppm (*m*, 2H), 2.17 ppm (*m*, 4H), 7.29–7.31 ppm (*d*, 1H), 8.11–8.12 ppm (*d*, 1H), 8.45 ppm (*s*, 1H), 3.39 ppm (*m*, 3H, CH_3_), 4.39 ppm (*m*, 2H, OH), 1.05 ppm (*m*, 6H, CH_3_), 3.43 ppm (*m*, 4H, CH_2_).

## 6. Refinement

Crystal data, data collection and structure refinement details are summarized in Table 4 ▸. Non-coordinated ethanol mol­ecules forming hydrogen bonds with the coordination fragment are disordered at two positions H9–O9*A*–C17*A*–C18*A* with an occupancy ratio of 0.8 and 0.2 for H9–O9*B*–C17*B*–C18*B*. Both disordered mol­ecules were refined anisotropically, with certain constraints applied to bond lengths and the same *U* ^ij^ components in the minor constituent. C-bound H atoms were positioned geometrically (C—H = 0.95–0.99 Å) and refined as riding with *U*_iso_(H) = 1.2*U*_eq_(C).

## 7. Thermogravimetric analysis

**Figure 5:**
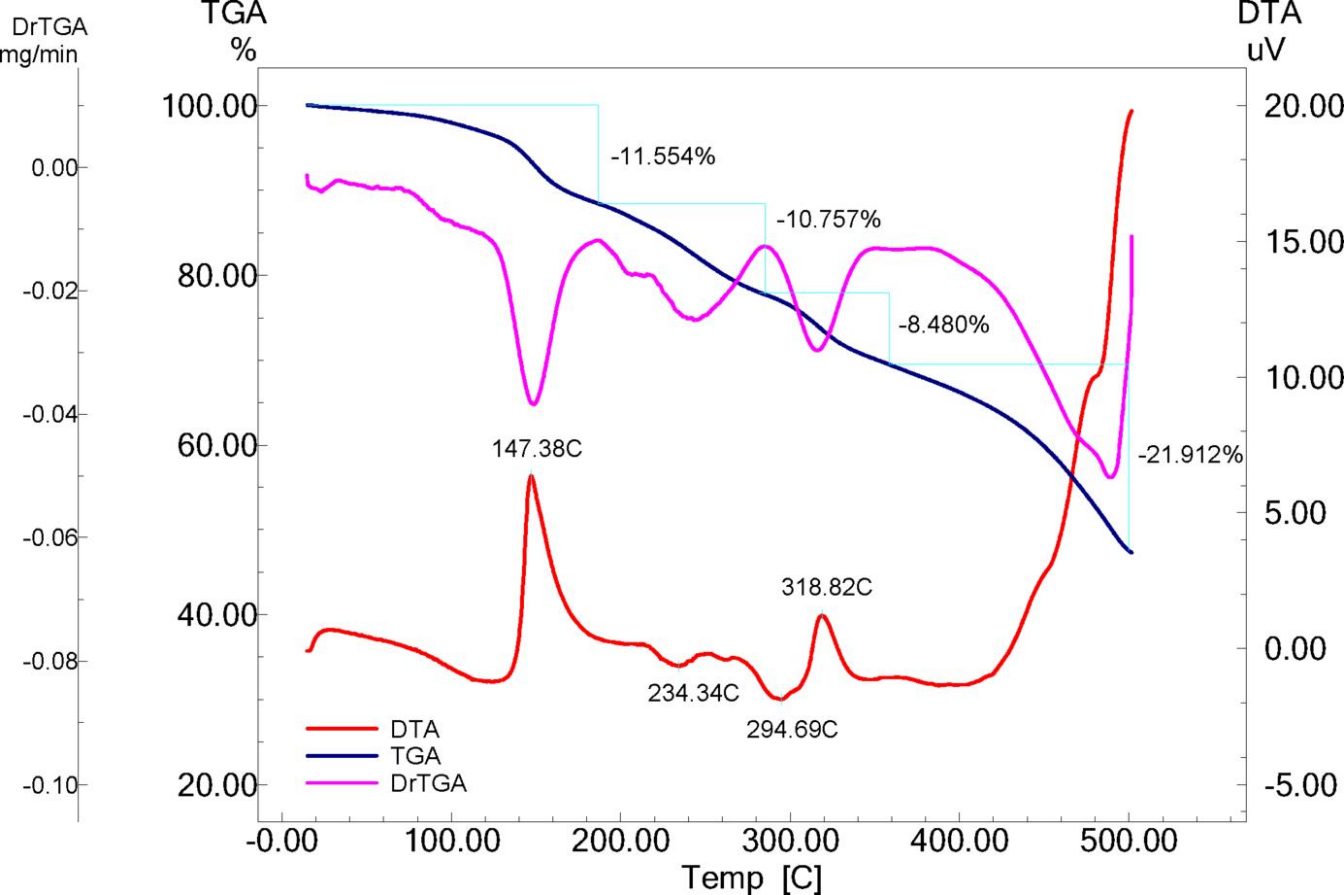
The DTA (red line), DrTGA (pink line) and TGA (blue line) weight loss trace for the sodium salt of mesotrione.

Four different stages of decomposition of the mesotrione-based sodium complex were observed in the investigated temperature range (Fig. 5 ▸). The first stage of thermal decomposition is characterized by a distinct exothermic effect and a mass loss of ∼12% in the temperature range of 25–182°C. The exothermic effect is observed at a temperature of 147°C (m.p. = 149–151°C), corresponding to the loss of the first ethanol mol­ecule.

At the second stage of the decomposition of the coordination compound in the temperature range 182–281°C, the loss (∼11%) of the second ethanol mol­ecule occurs, which is accompanied by an endothermic effect. The third stage of thermal decomposition is characterized by exothermic effect and a mass loss of ∼8.5% in the temperature range 280–340°C. The exothermic effect is observed at a temperature of 318.8°C, corresponding to the combustion of the entire organic components.

The fourth stage begins at 500°C and ends at 600°C and cannot be detected by the Shimadzu DTG-60H.

The TGV analysis and calculations based on its results show that the third and fourth stages consist of the combustion of the entire organic component of the mol­ecule and the formation of sodium pyro­sulfate.

**Figure 6:**
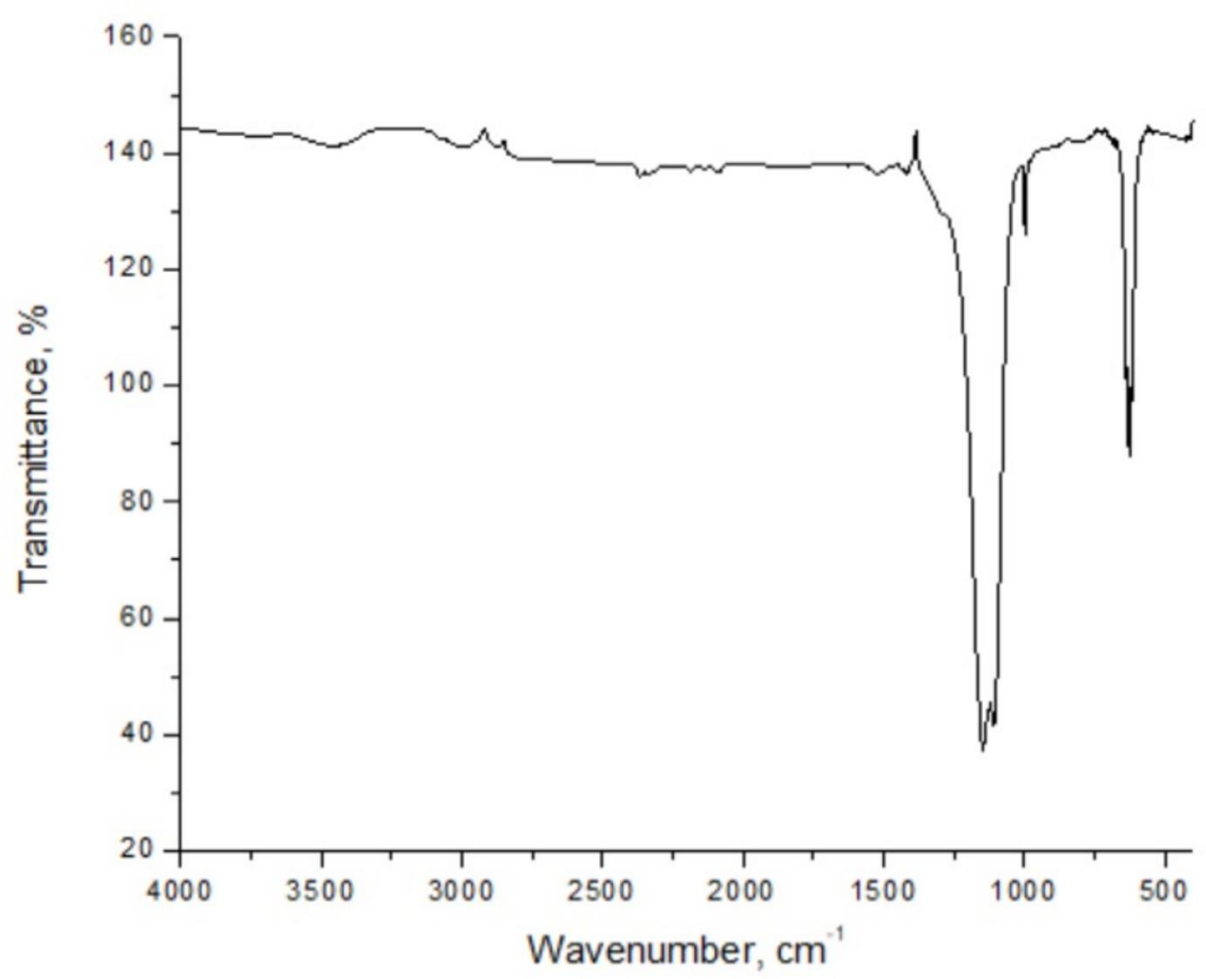
The IR spectrum for the final product after TGA (Na_2_SO_4_).

According to the thermal studies, the fourth stage is accompanied by a strong exothermic effect and includes the further transformation of Na_2_S_2_O_7_ into Na_2_SO_4_, which is confirmed by the results of IR spectroscopy (Fig. 6 ▸).

## Supplementary Material

Crystal structure: contains datablock(s) I. DOI: 10.1107/S2056989024001439/oi2003sup1.cif

Structure factors: contains datablock(s) I. DOI: 10.1107/S2056989024001439/oi2003Isup2.hkl

CCDC reference: 2072869

Additional supporting information:  crystallographic information; 3D view; checkCIF report

## Figures and Tables

**Table 1:** Selected geometric parameters (Å, °)

Na1—O6	2.2815 (17)	O3—N1	1.218 (3)
Na1—O5	2.3191 (18)	O4—N1	1.218 (2)
Na1—O5^i^	2.3215 (17)	O5—C14	1.251 (3)
Na1—O8	2.347 (2)	O6—C8	1.237 (3)
Na1—O1^ii^	2.3700 (19)	O7—C10	1.245 (3)
Na1—Na1^i^	3.3927 (18)	O8—C15	1.443 (3)
S1—O2	1.4386 (18)	N1—C3	1.466 (3)
S1—O1	1.4445 (18)	C4—C8	1.528 (3)
S1—C7	1.754 (3)	C8—C9	1.440 (3)
S1—C1	1.773 (2)	C9—C14	1.442 (3)
			
O6—Na1—O5	73.86 (6)	O1^ii^—Na1—Na1^i^	120.78 (6)
O6—Na1—O5^i^	159.89 (7)	O2—S1—O1	118.31 (11)
O5—Na1—O5^i^	86.04 (6)	O2—S1—C7	108.75 (13)
O6—Na1—O8	93.06 (7)	O1—S1—C7	108.40 (12)
O5—Na1—O8	122.94 (7)	S1—O1—Na1^ii^	144.85 (11)
O5^i^—Na1—O8	98.52 (7)	C14—O5—Na1	136.71 (15)
O6—Na1—O1^ii^	90.74 (7)	C14—O5—Na1^i^	129.29 (15)
O5—Na1—O1^ii^	124.59 (7)	Na1—O5—Na1^i^	93.96 (6)
O5^i^—Na1—O1^ii^	100.42 (7)	C8—O6—Na1	136.56 (15)
O8—Na1—O1^ii^	110.49 (7)	C15—O8—Na1	109.47 (15)
O6—Na1—Na1^i^	116.90 (6)	O3—N1—O4	123.5 (2)
O5—Na1—Na1^i^	43.05 (4)	O3—N1—C3	118.18 (19)
O5^i^—Na1—Na1^i^	42.99 (4)	O4—N1—C3	118.3 (2)
O8—Na1—Na1^i^	118.24 (6)		

Symmetry codes: (i) 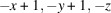 ; (ii) 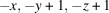 .

**Table 2:** Comparison between some geometrical parameters (Å) in the chelate ring for H*L* and Na*L* Note that the numbering of atoms in the H*L* structure has brought into accordance with the numbering in the published structure.

Bond	Na*L*	H*L*	*Δ*
C14—O5	1.252 (3)	1.314 (2)	0.062
C9—C14	1.442 (3)	1.382 (2)	0.06
C8—C9	1.439 (3)	1.448 (2)	0.009
C8—O6	1.237 (3)	1.239 (2)	0.02
C4—C8	1.528 (3)	1.514 (2)	0.014

**Table 3:** Hydrogen-bond geometry (Å, °)

*D*—H⋯*A*	*D*—H	H⋯*A*	*D*⋯*A*	*D*—H⋯*A*
O9*A*—H9⋯O8	0.90 (4)	1.98 (4)	2.875 (5)	170 (4)
O9*B*—H9⋯O8	0.91 (4)	1.98 (4)	2.81 (2)	152 (4)
O8—H8⋯O7^iii^	0.76 (3)	1.92 (3)	2.681 (2)	171 (3)
C2—H2⋯O6^ii^	0.95	2.59	3.229 (3)	125
C7—H7*B*⋯O4^ii^	0.98	2.43	3.200 (3)	135
C7—H7*C*⋯O9*A* ^iv^	0.98	2.37	3.349 (5)	176

Symmetry codes: (ii) 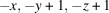 ; (iii) 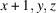 ; (iv) 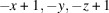 .

**Table 4:** Experimental details

Crystal data	
Chemical formula	[Na(C_14_H_12_NO_7_S)]·C_2_H_6_O
*M*_r_	453.43
Crystal system, space group	Triclinic, *P* 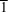
Temperature (K)	173
*a*, *b*, *c* (Å)	9.9014 (5), 10.7214 (6), 11.9401 (6)
α, β, γ (°)	69.789 (3), 71.074 (3), 66.439 (3)
*V* (Å^3^)	1064.45 (10)
*Z*	2
Radiation type	Mo *K*α
μ (mm^−1^)	0.22
Crystal size (mm)	0.36 × 0.23 × 0.18

Data collection	
Diffractometer	Bruker APEXII CCD
Absorption correction	Multi-scan (*SADABS*; Krause *et al.*, 2015 ▸)
*T*_min_, *T*_max_	0.679, 0.745
No. of measured, independent and observed [*I* > 2σ(*I*)] reflections	15328, 4340, 3259
*R*_int_	0.039
(sin θ/λ)_max_ (Å^−1^)	0.625

Refinement	
*R*[*F* ^2^ > 2σ(*F* ^2^)], *wR*(*F* ^2^), *S*	0.050, 0.133, 1.05
No. of reflections	4340
No. of parameters	308
No. of restraints	22
H-atom treatment	H atoms treated by a mixture of independent and constrained refinement
Δρ_max_, Δρ_min_ (e Å^−3^)	0.47, −0.41

Computer programs: *APEX2* and *SAINT* (Bruker, 2012 ▸), *SHELXS* and *SHELXTL* (Sheldrick, 2008 ▸) and *SHELXL* (Sheldrick, 2015 ▸).

## supplementary crystallographic information

### Crystal data

**Table:**

[Na(C_14_H_12_NO_7_S)]·C_2_H_6_O	*Z* = 2
*M**_r_* = 453.43	*F*(000) = 476
Triclinic, *P*1	*D*_x_ = 1.415 Mg m^−^^3^
*a* = 9.9014 (5) Å	Mo *K*α radiation, λ = 0.71073 Å
*b* = 10.7214 (6) Å	Cell parameters from 4340 reflections
*c* = 11.9401 (6) Å	θ = 1.9–26.4°
α = 69.789 (3)°	µ = 0.22 mm^−^^1^
β = 71.074 (3)°	*T* = 173 K
γ = 66.439 (3)°	Prizm, yellow
*V* = 1064.45 (10) Å^3^	0.36 × 0.23 × 0.18 mm

### Data collection

**Table:**

Bruker APEXII CCD diffractometer	3259 reflections with *I* > 2σ(*I*)
Radiation source: sealed tube	*R*_int_ = 0.039
φ and ω scans	θ_max_ = 26.4°, θ_min_ = 1.9°
Absorption correction: multi-scan (SADABS; Krause *et al.*, 2015)	*h* = −12→12
*T*_min_ = 0.679, *T*_max_ = 0.745	*k* = −13→10
15328 measured reflections	*l* = −14→14
4340 independent reflections	

### Refinement

**Table:**

Refinement on *F*^2^	22 restraints
Least-squares matrix: full	Hydrogen site location: mixed
*R*[*F*^2^ > 2σ(*F*^2^)] = 0.050	H atoms treated by a mixture of independent and constrained refinement
*wR*(*F*^2^) = 0.133	*w* = 1/[σ^2^(*F*_o_^2^) + (0.0686*P*)^2^ + 0.3718*P*] where *P* = (*F*_o_^2^ + 2*F*_c_^2^)/3
*S* = 1.05	(Δ/σ)_max_ < 0.001
4340 reflections	Δρ_max_ = 0.47 e Å^−^^3^
308 parameters	Δρ_min_ = −0.41 e Å^−^^3^

### Special details

**Table:**

Geometry. All esds (except the esd in the dihedral angle between two l.s. planes) are estimated using the full covariance matrix. The cell esds are taken into account individually in the estimation of esds in distances, angles and torsion angles; correlations between esds in cell parameters are only used when they are defined by crystal symmetry. An approximate (isotropic) treatment of cell esds is used for estimating esds involving l.s. planes.

### Fractional atomic coordinates and isotropic or equivalent isotropic displacement parameters (Å^2^)

**Table:**

	*x*	*y*	*z*	*U*_iso_*/*U*_eq_	Occ. (<1)
Na1	0.46287 (10)	0.49578 (10)	0.15007 (8)	0.0271 (2)	
S1	−0.20329 (6)	0.18062 (6)	0.76443 (5)	0.02386 (17)	
O1	−0.34311 (18)	0.29415 (18)	0.78282 (15)	0.0337 (4)	
O2	−0.2105 (2)	0.04798 (18)	0.76771 (16)	0.0361 (5)	
O3	−0.2679 (2)	0.6168 (2)	0.3932 (2)	0.0674 (8)	
O4	−0.0518 (2)	0.60798 (18)	0.27209 (15)	0.0346 (4)	
O5	0.37328 (18)	0.43201 (18)	0.02826 (14)	0.0295 (4)	
O6	0.27562 (18)	0.4081 (2)	0.27717 (15)	0.0317 (4)	
O7	−0.07874 (17)	0.3379 (2)	0.23648 (15)	0.0315 (4)	
O8	0.6424 (2)	0.3351 (2)	0.26488 (16)	0.0312 (4)	
H8	0.723 (4)	0.338 (3)	0.249 (3)	0.046 (10)*	
N1	−0.1331 (2)	0.5579 (2)	0.36258 (18)	0.0288 (5)	
C1	−0.0937 (2)	0.2401 (2)	0.6198 (2)	0.0201 (5)	
C2	−0.1495 (2)	0.3751 (2)	0.5520 (2)	0.0204 (5)	
H2	−0.243164	0.437557	0.583606	0.025*	
C3	−0.0661 (2)	0.4176 (2)	0.4367 (2)	0.0198 (5)	
C4	0.0718 (2)	0.3301 (2)	0.38681 (19)	0.0212 (5)	
C5	0.1247 (3)	0.1941 (3)	0.4576 (2)	0.0331 (6)	
H5	0.218752	0.131642	0.426409	0.040*	
C6	0.0421 (3)	0.1481 (3)	0.5734 (2)	0.0310 (6)	
H6	0.078492	0.054489	0.620104	0.037*	
C7	−0.1024 (3)	0.1529 (3)	0.8723 (2)	0.0360 (6)	
H7A	−0.155609	0.115410	0.954261	0.054*	
H7B	−0.093852	0.242129	0.868964	0.054*	
H7C	−0.001251	0.085614	0.853847	0.054*	
C8	0.1760 (2)	0.3745 (2)	0.2656 (2)	0.0228 (5)	
C9	0.1652 (2)	0.3566 (2)	0.1551 (2)	0.0214 (5)	
C10	0.0378 (3)	0.3228 (2)	0.1541 (2)	0.0226 (5)	
C11	0.0423 (3)	0.2744 (3)	0.0483 (2)	0.0257 (5)	
H11A	−0.016765	0.354371	−0.007590	0.031*	
H11B	−0.006094	0.200559	0.079509	0.031*	
C12	0.2020 (3)	0.2168 (3)	−0.0229 (2)	0.0276 (5)	
H12A	0.198851	0.194847	−0.095847	0.033*	
H12B	0.258493	0.129274	0.028904	0.033*	
C13	0.2798 (3)	0.3266 (3)	−0.0617 (2)	0.0277 (5)	
H13A	0.385898	0.286890	−0.103136	0.033*	
H13B	0.229579	0.408757	−0.121612	0.033*	
C14	0.2778 (2)	0.3753 (2)	0.0438 (2)	0.0222 (5)	
C15	0.5810 (3)	0.3345 (3)	0.3924 (2)	0.0428 (7)	
H15A	0.471176	0.351126	0.410164	0.051*	
H15B	0.594531	0.413466	0.407564	0.051*	
C16	0.6491 (4)	0.2045 (4)	0.4767 (3)	0.0584 (9)	
H16A	0.601652	0.212192	0.560994	0.088*	
H16B	0.633868	0.125845	0.464123	0.088*	
H16C	0.757414	0.188282	0.461518	0.088*	
O9A	0.7557 (4)	0.0676 (4)	0.2067 (4)	0.0470 (10)	0.815 (5)
C17A	0.6460 (4)	−0.0028 (4)	0.2653 (3)	0.0462 (11)	0.815 (5)
H17B	0.603839	0.007405	0.350234	0.055*	0.815 (5)
H17A	0.695169	−0.104216	0.268723	0.055*	0.815 (5)
C18A	0.5179 (5)	0.0571 (5)	0.1970 (5)	0.0668 (14)	0.815 (5)
H18C	0.444488	0.006821	0.239201	0.100*	0.815 (5)
H18B	0.467983	0.157119	0.194744	0.100*	0.815 (5)
H18A	0.559228	0.045602	0.113309	0.100*	0.815 (5)
O9B	0.7192 (18)	0.101 (2)	0.174 (2)	0.051 (4)	0.185 (5)
C17B	0.5847 (18)	0.0752 (18)	0.1820 (15)	0.045 (2)	0.185 (5)
H17C	0.601398	0.030955	0.116176	0.054*	0.185 (5)
H17D	0.501848	0.165750	0.169959	0.054*	0.185 (5)
C18B	0.538 (2)	−0.021 (2)	0.3067 (16)	0.080 (4)	0.185 (5)
H18D	0.445848	−0.036455	0.309274	0.120*	0.185 (5)
H18E	0.619223	−0.111630	0.318195	0.120*	0.185 (5)
H18F	0.519738	0.023078	0.371944	0.120*	0.185 (5)
H9	0.709 (4)	0.153 (4)	0.223 (4)	0.078 (13)*	

### Atomic displacement parameters (Å^2^)

**Table:**

	*U*^11^	*U*^22^	*U*^33^	*U*^12^	*U*^13^	*U*^23^
Na1	0.0234 (5)	0.0371 (6)	0.0197 (5)	−0.0147 (4)	−0.0002 (4)	−0.0042 (4)
S1	0.0249 (3)	0.0274 (3)	0.0163 (3)	−0.0136 (3)	−0.0001 (2)	0.0001 (2)
O1	0.0266 (9)	0.0376 (11)	0.0238 (9)	−0.0107 (8)	0.0063 (7)	−0.0027 (8)
O2	0.0493 (11)	0.0315 (10)	0.0288 (10)	−0.0248 (9)	−0.0026 (8)	−0.0005 (8)
O3	0.0473 (14)	0.0426 (13)	0.0484 (14)	0.0102 (10)	0.0132 (11)	0.0123 (10)
O4	0.0408 (11)	0.0342 (10)	0.0242 (9)	−0.0214 (9)	−0.0043 (8)	0.0066 (8)
O5	0.0272 (9)	0.0438 (11)	0.0191 (9)	−0.0216 (8)	0.0012 (7)	−0.0036 (8)
O6	0.0289 (9)	0.0544 (12)	0.0191 (9)	−0.0259 (9)	−0.0009 (7)	−0.0072 (8)
O7	0.0196 (9)	0.0551 (12)	0.0245 (9)	−0.0200 (8)	0.0034 (7)	−0.0137 (8)
O8	0.0224 (10)	0.0427 (11)	0.0277 (10)	−0.0150 (8)	−0.0043 (8)	−0.0035 (8)
N1	0.0326 (12)	0.0253 (11)	0.0214 (11)	−0.0096 (10)	−0.0015 (9)	−0.0015 (9)
C1	0.0213 (12)	0.0264 (13)	0.0138 (11)	−0.0117 (10)	−0.0014 (9)	−0.0038 (9)
C2	0.0181 (11)	0.0241 (13)	0.0179 (11)	−0.0072 (10)	−0.0001 (9)	−0.0070 (10)
C3	0.0226 (12)	0.0209 (12)	0.0173 (11)	−0.0099 (10)	−0.0054 (9)	−0.0019 (9)
C4	0.0199 (12)	0.0302 (13)	0.0138 (11)	−0.0119 (10)	−0.0029 (9)	−0.0020 (10)
C5	0.0224 (13)	0.0358 (15)	0.0222 (13)	−0.0007 (11)	0.0027 (10)	−0.0026 (11)
C6	0.0276 (13)	0.0248 (13)	0.0237 (13)	−0.0031 (11)	−0.0025 (10)	0.0044 (11)
C7	0.0393 (16)	0.0509 (18)	0.0184 (13)	−0.0244 (14)	−0.0039 (11)	0.0001 (12)
C8	0.0180 (11)	0.0279 (13)	0.0176 (12)	−0.0083 (10)	−0.0021 (9)	−0.0006 (10)
C9	0.0182 (11)	0.0284 (13)	0.0156 (11)	−0.0101 (10)	−0.0011 (9)	−0.0022 (10)
C10	0.0213 (12)	0.0235 (12)	0.0195 (12)	−0.0078 (10)	−0.0054 (10)	0.0004 (10)
C11	0.0251 (13)	0.0313 (14)	0.0237 (13)	−0.0139 (11)	−0.0043 (10)	−0.0057 (11)
C12	0.0297 (13)	0.0288 (14)	0.0231 (13)	−0.0094 (11)	−0.0042 (10)	−0.0064 (11)
C13	0.0276 (13)	0.0368 (15)	0.0164 (12)	−0.0149 (11)	0.0007 (10)	−0.0039 (10)
C14	0.0188 (11)	0.0264 (13)	0.0165 (11)	−0.0069 (10)	−0.0048 (9)	0.0009 (10)
C15	0.0311 (15)	0.060 (2)	0.0326 (16)	−0.0143 (14)	−0.0024 (12)	−0.0106 (14)
C16	0.067 (2)	0.065 (2)	0.046 (2)	−0.0285 (19)	−0.0158 (17)	−0.0064 (17)
O9A	0.034 (2)	0.042 (2)	0.059 (3)	−0.0073 (17)	−0.0052 (15)	−0.0159 (18)
C17A	0.056 (3)	0.037 (2)	0.044 (2)	−0.0167 (18)	−0.0024 (18)	−0.0139 (17)
C18A	0.047 (3)	0.063 (3)	0.090 (4)	−0.023 (2)	−0.009 (3)	−0.017 (3)
O9B	0.034 (6)	0.048 (6)	0.057 (7)	−0.005 (5)	0.001 (5)	−0.017 (5)
C17B	0.056 (4)	0.037 (4)	0.045 (4)	−0.013 (3)	−0.006 (3)	−0.020 (3)
C18B	0.052 (6)	0.075 (6)	0.097 (7)	−0.015 (6)	−0.010 (6)	−0.015 (6)

### Geometric parameters (Å, º)

**Table:**

Na1—O6	2.2815 (17)	C9—C10	1.449 (3)
Na1—O5	2.3191 (18)	C10—C11	1.504 (3)
Na1—O5^i^	2.3215 (17)	C11—C12	1.521 (3)
Na1—O8	2.347 (2)	C11—H11A	0.9900
Na1—O1^ii^	2.3699 (19)	C11—H11B	0.9900
Na1—Na1^i^	3.3928 (18)	C12—C13	1.520 (3)
S1—O2	1.4386 (18)	C12—H12A	0.9900
S1—O1	1.4445 (18)	C12—H12B	0.9900
S1—C7	1.754 (3)	C13—C14	1.514 (3)
S1—C1	1.773 (2)	C13—H13A	0.9900
O3—N1	1.218 (3)	C13—H13B	0.9900
O4—N1	1.218 (2)	C15—C16	1.459 (4)
O5—C14	1.251 (3)	C15—H15A	0.9900
O6—C8	1.237 (3)	C15—H15B	0.9900
O7—C10	1.245 (3)	C16—H16A	0.9800
O8—C15	1.443 (3)	C16—H16B	0.9800
O8—H8	0.76 (3)	C16—H16C	0.9800
N1—C3	1.466 (3)	O9A—C17A	1.4270 (19)
C1—C2	1.377 (3)	O9A—H9	0.90 (4)
C1—C6	1.386 (3)	C17A—C18A	1.531 (2)
C2—C3	1.384 (3)	C17A—H17B	0.9900
C2—H2	0.9500	C17A—H17A	0.9900
C3—C4	1.390 (3)	C18A—H18C	0.9800
C4—C5	1.393 (3)	C18A—H18B	0.9800
C4—C8	1.528 (3)	C18A—H18A	0.9800
C5—C6	1.393 (3)	O9B—C17B	1.429 (2)
C5—H5	0.9500	O9B—H9	0.91 (4)
C6—H6	0.9500	C17B—C18B	1.539 (2)
C7—H7A	0.9800	C17B—H17C	0.9900
C7—H7B	0.9800	C17B—H17D	0.9900
C7—H7C	0.9800	C18B—H18D	0.9800
C8—C9	1.440 (3)	C18B—H18E	0.9800
C9—C14	1.442 (3)	C18B—H18F	0.9800
			
O6—Na1—O5	73.86 (6)	O7—C10—C9	121.8 (2)
O6—Na1—O5^i^	159.89 (7)	O7—C10—C11	118.6 (2)
O5—Na1—O5^i^	86.04 (6)	C9—C10—C11	119.58 (19)
O6—Na1—O8	93.06 (7)	C10—C11—C12	112.88 (19)
O5—Na1—O8	122.94 (7)	C10—C11—H11A	109.0
O5^i^—Na1—O8	98.52 (7)	C12—C11—H11A	109.0
O6—Na1—O1^ii^	90.74 (7)	C10—C11—H11B	109.0
O5—Na1—O1^ii^	124.59 (7)	C12—C11—H11B	109.0
O5^i^—Na1—O1^ii^	100.41 (7)	H11A—C11—H11B	107.8
O8—Na1—O1^ii^	110.49 (7)	C13—C12—C11	108.7 (2)
O6—Na1—Na1^i^	116.90 (6)	C13—C12—H12A	109.9
O5—Na1—Na1^i^	43.05 (4)	C11—C12—H12A	109.9
O5^i^—Na1—Na1^i^	42.99 (4)	C13—C12—H12B	109.9
O8—Na1—Na1^i^	118.24 (6)	C11—C12—H12B	109.9
O1^ii^—Na1—Na1^i^	120.78 (6)	H12A—C12—H12B	108.3
O2—S1—O1	118.31 (11)	C14—C13—C12	113.39 (19)
O2—S1—C7	108.75 (13)	C14—C13—H13A	108.9
O1—S1—C7	108.40 (12)	C12—C13—H13A	108.9
O2—S1—C1	107.70 (10)	C14—C13—H13B	108.9
O1—S1—C1	107.16 (11)	C12—C13—H13B	108.9
C7—S1—C1	105.83 (11)	H13A—C13—H13B	107.7
S1—O1—Na1^ii^	144.85 (11)	O5—C14—C9	123.8 (2)
C14—O5—Na1	136.71 (15)	O5—C14—C13	117.54 (19)
C14—O5—Na1^i^	129.29 (15)	C9—C14—C13	118.67 (19)
Na1—O5—Na1^i^	93.96 (6)	O8—C15—C16	114.3 (3)
C8—O6—Na1	136.56 (15)	O8—C15—H15A	108.7
C15—O8—Na1	109.47 (15)	C16—C15—H15A	108.7
C15—O8—H8	108 (2)	O8—C15—H15B	108.7
Na1—O8—H8	122 (2)	C16—C15—H15B	108.7
O3—N1—O4	123.5 (2)	H15A—C15—H15B	107.6
O3—N1—C3	118.18 (19)	C15—C16—H16A	109.5
O4—N1—C3	118.3 (2)	C15—C16—H16B	109.5
C2—C1—C6	120.9 (2)	H16A—C16—H16B	109.5
C2—C1—S1	119.19 (17)	C15—C16—H16C	109.5
C6—C1—S1	119.77 (18)	H16A—C16—H16C	109.5
C1—C2—C3	118.5 (2)	H16B—C16—H16C	109.5
C1—C2—H2	120.8	C17A—O9A—H9	104 (2)
C3—C2—H2	120.8	O9A—C17A—C18A	111.3 (4)
C2—C3—C4	122.8 (2)	O9A—C17A—H17B	109.4
C2—C3—N1	117.48 (19)	C18A—C17A—H17B	109.4
C4—C3—N1	119.59 (19)	O9A—C17A—H17A	109.4
C3—C4—C5	117.3 (2)	C18A—C17A—H17A	109.4
C3—C4—C8	125.3 (2)	H17B—C17A—H17A	108.0
C5—C4—C8	117.2 (2)	C17A—C18A—H18C	109.5
C4—C5—C6	121.1 (2)	C17A—C18A—H18B	109.5
C4—C5—H5	119.5	H18C—C18A—H18B	109.5
C6—C5—H5	119.5	C17A—C18A—H18A	109.5
C1—C6—C5	119.4 (2)	H18C—C18A—H18A	109.5
C1—C6—H6	120.3	H18B—C18A—H18A	109.5
C5—C6—H6	120.3	C17B—O9B—H9	115 (3)
S1—C7—H7A	109.5	O9B—C17B—C18B	111.6 (17)
S1—C7—H7B	109.5	O9B—C17B—H17C	109.3
H7A—C7—H7B	109.5	C18B—C17B—H17C	109.3
S1—C7—H7C	109.5	O9B—C17B—H17D	109.3
H7A—C7—H7C	109.5	C18B—C17B—H17D	109.3
H7B—C7—H7C	109.5	H17C—C17B—H17D	108.0
O6—C8—C9	126.1 (2)	C17B—C18B—H18D	109.5
O6—C8—C4	113.4 (2)	C17B—C18B—H18E	109.5
C9—C8—C4	119.87 (19)	H18D—C18B—H18E	109.5
C8—C9—C14	120.88 (19)	C17B—C18B—H18F	109.5
C8—C9—C10	119.83 (19)	H18D—C18B—H18F	109.5
C14—C9—C10	119.3 (2)	H18E—C18B—H18F	109.5

Symmetry codes: (i) −*x*+1, −*y*+1, −*z*; (ii) −*x*, −*y*+1, −*z*+1.

### Hydrogen-bond geometry (Å, º)

**Table:**

*D*—H···*A*	*D*—H	H···*A*	*D*···*A*	*D*—H···*A*
O9*A*—H9···O8	0.90 (4)	1.98 (4)	2.875 (5)	170 (4)
O9*B*—H9···O8	0.91 (4)	1.98 (4)	2.81 (2)	152 (4)
O8—H8···O7^iii^	0.76 (3)	1.92 (3)	2.681 (2)	171 (3)
C2—H2···O6^ii^	0.95	2.59	3.229 (3)	125
C7—H7*B*···O4^ii^	0.98	2.43	3.200 (3)	135
C7—H7*C*···O9*A*^iv^	0.98	2.37	3.349 (5)	176

Symmetry codes: (ii) −*x*, −*y*+1, −*z*+1; (iii) *x*+1, *y*, *z*; (iv) −*x*+1, −*y*, −*z*+1.
